# Occupational Therapy Consultation in Out-of-School-Time Care (Gakudo-Hoiku): A Case Report on Supporting a Child With Impulsive Behavior and Enhancing Staff Partnership

**DOI:** 10.7759/cureus.105711

**Published:** 2026-03-23

**Authors:** Megumi Ogiwara-Hirano, Takayuki Yaegashi, Saeko Takenaka, Hiromi Fujii

**Affiliations:** 1 Operations, Linie R, Inc., Tokyo, JPN; 2 Occupational Therapy, Graduate School, Yamagata Prefectural University of Health Sciences, Yamagata, JPN

**Keywords:** collaborative consultation, inclusive environment, occupational therapy consultation, out-of-school-time care (gakudo-hoiku), peo model

## Abstract

This case report aimed to clarify the intervention process and effectiveness of occupational therapist (OT) consultation in Japanese out-of-school-time childcare (Gakudo-Hoiku). The intervention focused on a school-aged girl who exhibited impulsive outdoor bolting and difficulty remaining indoors due to sensory-seeking behaviors.

The participant was a third-grade elementary school girl without a formal medical or developmental diagnosis. The intervention was conducted from April 2023 to January 2024, with monthly consultation visits lasting approximately four hours each. The OT used a collaborative consultation approach that included explaining the child’s developmental and behavioral characteristics to childcare staff, proposing physical environmental modifications (e.g., installation of auxiliary door locks), and grading meaningful activities by assigning structured roles aligned with the child’s interests, such as assisting with picture-story shows (kamishibai).

Physical environmental modifications were associated with a marked reduction in unannounced outdoor bolting, substantially decreasing the supervisory burden on staff. In addition, assigning roles based on the child’s interests promoted proactive participation during closing assemblies and facilitated positive interactions with peers.

By functioning as a “clinical interpreter of behavioral characteristics” and an “environmental mediator” between the child and staff, the OT enhanced staff self-efficacy and contributed to the development of a sustainable support system within the childcare setting. OT consultation in Gakudo-Hoiku may play an important role in fostering inclusive environments and supporting the well-being of children with diverse developmental needs.

## Introduction

After-school healthy nurturing programs in Japan (hereafter referred to as Gakudo-Hoiku) are defined under Article 6-3, Paragraph 2, of the Child Welfare Act as services that provide appropriate opportunities for play and daily living for elementary school children whose guardians are absent during the daytime due to work or other reasons [1]. These programs are categorized as “Type II Social Welfare Services” under the Social Welfare Act and are therefore required to notify local governments and ensure appropriate operational management [2]. In April 2025, the Operational Guidelines for After-school Children’s Clubs, which serve as the foundational framework for Gakudo-Hoiku practice, were substantially revised and subsequently implemented [3]. The revised guidelines place greater emphasis on respect for children’s rights, the strengthening of sexual violence prevention measures, safety management and business continuity planning, and collaboration with relevant organizations.

In recent years, demand for Gakudo-Hoiku services has increased rapidly, largely due to the rising number of dual-income households in Japan. A national survey conducted in July 2025 reported that the number of children using these services reached a record high of 1,568,588, representing an increase of 48,636 compared with the previous year [4]. As a result, approximately 17,000 children remain on waiting lists, particularly in urban areas, and the so-called “Grade 4 Wall,” which refers to the shortage of facilities capable of accommodating older elementary school children, continues to pose a serious challenge. At the same time, improving the quality of care to ensure the creation of inclusive environments for children with developmental disabilities has become an urgent issue. This trend reflects a fundamental shift in the role of Gakudo-Hoiku, from a mere "place to stay" to a "crucial arena for promoting children's overall well-being and social participation," where occupational therapists (OTs) can play a significant role. In Okayama Prefecture, children with disabilities account for 8.68% of the total Gakudo-Hoiku population, and there is a strong demand from frontline staff for professional consultation regarding concrete and practical support strategies [5]. Furthermore, the need for specialized support extends beyond children with diagnosed disabilities. Childcare staff frequently encounter children who lack a formal medical diagnosis but exhibit significant sensory-seeking behaviors and impulsivity. In the dynamic and highly stimulating environment of after-school care, these characteristics often manifest as difficulties in self-regulation, unpredictable bolting, and peer conflicts. Recognizing these underlying sensory and behavioral needs to provide appropriate environmental adjustments has become a critical challenge in daily childcare operations.

In the United States, which has advanced systems for utilizing specialized professionals in after-school settings, the Every Student Succeeds Act designates OTs as “Specialized Instructional Support Personnel” [6,7]. Furthermore, under the Individuals with Disabilities Education Act, OTs provide “related services” that support children’s participation across educational environments, including out-of-school-time care. These services are commonly structured within a three-tiered framework: Tier 3, which focuses on individualized support; Tier 2, which targets small-group interventions; and Tier 1, which emphasizes universal support, including environmental modifications and staff consultation. Accordingly, a legal and institutional foundation exists in the United States for OTs to contribute to children’s health, safety, and participation across their entire daily living environment [8]. This tiered approach provides a valuable model for Japan, suggesting that OTs can not only provide individualized support (Tiers 2 and 3) but also enhance the capacity of Gakudo-Hoiku staff and the surrounding environment (Tier 1).

In Japan, initiatives involving OT consultation, where OTs visit Gakudo-Hoiku facilities to provide on-site support, have expanded nationwide since approximately 2016, led by organizations such as the Okayama Prefecture Gakudo-Hoiku Association. Nevertheless, many facilities continue to face persistent challenges, including shortages of specialized personnel and increased environmental stress associated with large group sizes. This paper reports a case in which OT consultation was implemented for a child exhibiting impulsive behaviors, including outdoor bolting and difficulty remaining indoors. The purpose of this study is to examine the outcomes of this OT consultation and to discuss the potential future roles of OTs in Gakudo-Hoiku settings.

This research was presented as a poster at the 58th Annual Meeting of the Japanese Occupational Therapy Association, held on November 9, 2024.

## Case presentation

Subject and ethical considerations

The participant was a nine-year-old, third-grade girl with no formal medical or developmental diagnosis. She was enrolled in a regular classroom at her elementary school and attended after-school Club A on a daily basis. In the school setting, she experienced difficulty remaining seated during lessons and frequently bolted toward the schoolyard. This study was approved by the Linie R Ethics Committee (Approval No. 2086) and conducted in accordance with the Declaration of Helsinki. Written informed consent for publication was obtained from the participant’s legal guardians.

Chief complaints and identified needs

Childcare staff reported multiple challenges, including unpredictable outdoor bolting and difficulty returning the child to the room following water play, destruction of other children’s work, verbal and physical aggression toward peers, and difficulties in providing effective support to the family. At the same time, staff expressed a strong desire to leverage the child’s strengths and interests in order to enable her to spend time in the facility safely and positively.

Observation and evaluation

Target Behaviors

The child demonstrated marked impulsivity and sensory-seeking tendencies, which were associated with limited self-regulation. During bolting episodes, multiple staff members were required to pursue her, thereby compromising supervision and safety for other children in the facility.

Social Interaction

Despite these challenges, the child showed enjoyment in caring for younger children and displayed positive rapport with specific female staff members.

Environmental Assessment

The facility served approximately 60 enrolled children, resulting in an environment characterized by high levels of auditory and visual stimulation.

Evaluation Process

Although formal standardized assessments were not performed, the child’s behavioral and sensory characteristics were meticulously documented through continuous informal clinical observations within her naturalistic daily environment. These findings were validated through integrated multidisciplinary perspectives, combining the clinical reasoning of the OT with the longitudinal observations of the childcare staff, ensuring a comprehensive understanding of her functional needs.

Implementation method and consultation framework

The intervention period extended from April 2023 to January 2024. Consultation visits were conducted once per month, with each visit lasting approximately four hours. To ensure intervention fidelity and reproducibility, each consultation session was systematically structured into a three-step collaborative framework.

Progress Review and Monitoring (Approximately 30-45 Minutes)

Each visit commenced with a review of the strategies implemented since the previous session. The OT and staff collaboratively evaluated the fidelity of the agreed-upon interventions (e.g., the consistent use of auxiliary locks and the Saturday footbath routine). Changes in the child’s target behaviors were monitored based on the staff’s daily anecdotal reports and shared reflections, allowing the team to assess the effectiveness of the ongoing support.

Onsite Observation and Activity Participation (Approximately 2 Hours)

The OT conducted naturalistic observations of the child during free play, structured activities, and environmental transitions. During this phase, the OT directly interacted with the child to assess her sensory processing and occupational performance.

Collaborative Reflection and Strategy Formulation (Approximately 1-1.5 Hours)

Following the observation, a joint case conference was held. The OT shared a clinical interpretation of the child’s behavior, translating sensory and developmental needs into accessible concepts for the staff. The OT explained to the childcare staff how to adjust verbal instructions and level out tasks. Together, the OT and staff co-created contextually appropriate, actionable strategies (e.g., assigning the Kamishibai role) for the upcoming month.

This iterative cycle of reviewing, observing, and collaborative planning ensured continuous monitoring of the recommendations and maintained high intervention fidelity throughout the 10-month period.

Course and intervention

Phase 1: Understanding Developmental Characteristics and Ensuring Safety Through Environmental Adjustment (April-July)

Assessment: The child exhibited pronounced impulsivity and emotional dysregulation, particularly during activity transitions. Outdoor bolting occurred frequently and often required the involvement of three staff members, which adversely affected the overall safety management of the group. In the home context, the child’s mother reported feelings of isolation, stemming from the father’s strict approach to parenting and nutrition, and expressed hesitation about seeking professional consultation.

Consultation: The OT provided staff with explanations regarding the developmental and sensory characteristics underlying the child’s behaviors. As a primary safety measure to prevent bolting, the OT proposed the installation of auxiliary door locks. The rationale for this recommendation was to interrupt the cycle of escalation and aggression that frequently occurred when staff attempted to physically force the child back indoors. In addition, the OT advised limiting the number of staff members responding to behavioral outbursts and recommended the use of clear, time-based verbal prompts to support transitions back into the room. To address the child’s need for sensory input and energy expenditure, the OT initially suggested the use of a trampoline; however, staff raised concerns regarding safety, limited space, and insufficient personnel. Respecting the staff’s strong emphasis on safety, the OT revised the intervention plan and explored alternative strategies in collaboration with the team.

Phase 2: Trial of Alternative Strategies and Collaboration With Staff (August-October)

Assessment: The frequency of outdoor bolting decreased markedly following the installation of auxiliary locks. Even when the child exited the room, the time required for her to return was substantially reduced. However, her strong sensory-seeking need for water-related play persisted, manifesting as repeated manipulation of faucets and damage to umbrellas when playing in puddles.

Consultation: To safely redirect the child’s interest in water, the OT proposed the introduction of a structured “footbath” activity. Although staff initially expressed concerns regarding potential messes and interpersonal conflicts, a system was collaboratively established in which the child assumed responsibility for preparation and cleanup. The activity was scheduled on Saturdays, when fewer children were present, in order to minimize staff burden while promoting the child’s initiative and sense of responsibility.

In addition, based on staff observations of the child’s engagement patterns, the OT suggested the use of card games as an alternative indoor activity. Throughout this phase, the OT intentionally adopted a stance of “learning from the staff,” acknowledging that their continuous, day-to-day observations were essential for designing effective indirect interventions.

Phase 3: Acquisition of Meaningful Roles and Proactive Participation (November Onward)

Assessment: Emotional outbursts and destructive behaviors decreased sharply during this phase. The child began to enjoy indoor activities with peers and demonstrated increased willingness to participate in group activities, including the closing meeting, which she had previously avoided.

Consultation: Based on observations of the child’s nurturing behavior toward a younger peer during card games, the OT proposed assigning her the role of a Kamishibai (paper drama) storyteller. She gradually began preparing the materials independently and reading stories aloud to her peers. Although she occasionally experienced difficulty with reading, she successfully completed the activity by collaborating with a peer who served as an “assistant.”

Building on the OT’s recommendations, staff introduced additional environmental modifications by placing a desk over the footbath area to reduce visual stimulation, thereby supporting the child’s ability to focus on origami and peer interaction. Furthermore, staff and children jointly created a sign and established shared “footbath rules” to guide participation in the activity. This integration of OT consultation and staff-driven creativity fostered the child’s spontaneous engagement within a safe, structured, and enjoyable environment.

Results

The installation of auxiliary door locks was associated with a clear reduction in outdoor bolting and a corresponding decrease in staff psychological burden. The environmental modification created a "safe space" that allowed the staff to shift their focus from crisis management to positive engagement. Through engagement in card games and the assignment of the Kamishibai storytelling role, the child developed cooperative and positive relationships with peers. By December 2023, the footbath activity, when combined with tabletop tasks and clearly visualized rules, enabled the child to participate for extended periods in a calm and relaxed manner. The intervention transformed her sensory-seeking behavior into meaningful social participation.

## Discussion

Staff working in after-school care settings frequently experience fatigue and may come to perceive children’s challenging behaviors primarily negatively. In this case, the OT initially functioned as an “interpreter of behavioral characteristics,” clarifying the underlying mechanisms of the child’s behaviors using principles from Applied Behavior Analysis and Sensory Integration theory, grounded in the Person-Environment-Occupation (PEO) model [9]. By translating the child’s sensory and behavioral needs into an evidence-based framework, the OT provided staff with a shared professional perspective. Developing this “common language” appeared to enhance staff confidence, improve the quality of care, and contribute to reducing occupational stress [10].

Building upon this shared understanding, the OT acted as an “adjuster of activities and the environment.” The OT capitalized on the child’s strengths and applied activity grading by systematically adjusting task difficulty. In addition, structuring the environment through the use of auxiliary locks, an approach emphasized in US school-based occupational therapy, helped ensure safety while simultaneously reducing the monitoring burden on staff [11]. Assigning the child the role of a Kamishibai storyteller further promoted occupational engagement, thereby transforming the Gakudo-Hoiku setting into a “fun place with a meaningful role.” These findings indicate that OT expertise is highly compatible with the dynamic and lived environment of after-school care.

Crucially, the core feature of this consultation was not the delivery of “professionally correct answers,” but rather the adoption of a collaborative consultation stance [12]. By jointly exploring contextually appropriate solutions, such as postponing trampoline use or restricting footbath activities to Saturdays, the OT enhanced staff members’ sense of self-efficacy [13]. Importantly, the true value of consultation lies in fostering sustainability, such that effective support can be maintained even after the OT’s involvement has ended.

Through these integrated roles, the OT functioned as a “lubricant,” facilitating adjustments among the child, staff, and the surrounding environment. The significance of this role can be summarized in four key aspects: (1) the realization of an inclusive environment through environmental modification; (2) the enhancement of staff expertise through the development of a shared professional language; (3) the promotion of skill generalization within a real-life context [14]; and (4) a conceptual shift from a medical model toward a social model of support. From this perspective, it is equally applicable to Child Developmental Support and After-school Day Care services, as well as home-based occupational therapy for school non-attendance [15,16]. Therefore, OT outreach to community-based settings is essential for promoting children’s overall well-being [17].

Despite these positive outcomes, several limitations must be acknowledged. This report is based on a single case, and therefore, the findings may not be broadly generalizable. Additionally, a key limitation of this study is the reliance on informal clinical observations rather than a standardized occupational therapy assessment template to establish a formal occupational diagnosis. Moreover, the observed success depended largely on the high level of motivation demonstrated by the staff. Future directions include the development of a standardized Japanese After-School OT Consultation Protocol using multicenter data. Such a protocol should incorporate formalized OT evaluation tools to enhance the objective measurement of outcomes and standardize the plan of care. Furthermore, quantitative validation employing outcome indicators, such as staff turnover rates and secondary effects on children’s school life, will be necessary to strengthen the evidence base.

## Conclusions

This case demonstrates that the role of an OT in Gakudo-Hoiku extends beyond “child training” to emphasize partnership building, in which staff members share expertise and collaboratively generate context-appropriate solutions. Through coordinated adjustments to the physical environment (e.g., locks), the social environment (e.g., staff interactions), and occupations (e.g., role assignment), the child’s difficulties in participation were effectively mitigated. As the demand for Gakudo-Hoiku continues to increase, OT outreach will play a crucial role in safeguarding children’s “place to belong” within after-school care settings.
